# Connectivity indices can predict population persistence in river networks: insights from a metapopulation model

**DOI:** 10.1007/s10980-025-02278-8

**Published:** 2026-01-08

**Authors:** Ali Gharouni, Richard Pither, Bronwyn Rayfield, David Cote, Frithjof Lutscher

**Affiliations:** 1 Department of Mathematics and Statistics, University of Ottawa, Ottawa, Canada; 2 Present Address: Ontario Public Health, Ottawa, Canada; 3 Landscape Science and Technology Division, Environment and Climate Change Canada, Ottawa, ON Canada; 4 Department of Biology, Carleton University, Ottawa, Canada; 5 Northwest Atlantic Fisheries Centre, Fisheries and Oceans Canada, St. John’s, NL Canada; 6 Department of Biology, University of Ottawa, Ottawa, Canada

**Keywords:** Aquatic connectivity, Freshwater fish, Colonization, Population indicators, Dendritic ecological network, Fish passage

## Abstract

**Context:**

Connectivity across river networks facilitates species movement and ecological processes that contribute to freshwater biodiversity. Certain indices provide measures of connectivity to focus conservation planning.

**Objectives:**

Our objective was to test whether commonly used connectivity indicators based on network structure can reliably predict population persistence.

**Methods:**

We used a spatially explicit metapopulation model for freshwater fish that complete their life cycle entirely within river networks and depend on connectivity for movement. Simulations were conducted across a range of network sizes, topologies, dispersal abilities, and barrier passabilities. We assessed the relationship between the Dendritic Connectivity Index (DCI) and metrics of persistence at the network and the reach scale.

**Results:**

DCI was strongly correlated with persistence at both the network and reach scale across most simulated network sizes and configurations, particularly in dendritic (branching) systems with symmetric barrier passability. At the network scale, correlations were strongest with density-independent persistence metrics, which is expected since DCI does not incorporate population interactions. Species dispersal ability influenced DCI–persistence correlations differently across scales: correlations were strongest at the network scale when dispersal distances spanned the full network (global dispersal) and at the reach scale when movement was limited to neighbouring segments (local dispersal). We also found that increases in DCI following simulated barrier removal were associated with improvements in persistence, further demonstrating its potential to support restoration efforts.

**Conclusion:**

Indicators like DCI can inform connectivity-focused conservation planning in river networks.

**Supplementary Information:**

The online version contains supplementary material available at 10.1007/s10980-025-02278-8.

## Introduction

Connectivity is a fundamental characteristic of flowing-water ecosystems, including streams and rivers, hereafter referred to as ‘rivers’ following McIntosh et al. (2024). These river networks are shaped by their dendritic branching structure, and their connectivity influences the movement of water, sediment, nutrients, and species that shape ecological processes (Torgersen et al. 2022; Ward 1989). River connectivity functions across multiple dimensions: longitudinal (along the river channel), lateral (between the river and floodplain), vertical (to groundwater and atmosphere), and temporal (seasonal or perennial flow) (Grill et al. 2019; Kondolf et al. 2006). Human-made barriers disrupt these natural flows, causing connectivity loss at multiple scales, from localized disruptions at individual river reaches to cumulative impacts across entire river networks. This loss of connectivity can reduce the abundance and diversity of riverine taxa, alter population structure, and drive long-term evolutionary changes (Ardren and Bernall 2017; Barbarossa et al. 2020; Fuller et al. 2015; Thieme et al. 2024; Zarri et al. 2022). As the ecological and evolutionary consequences of connectivity loss become apparent, global efforts to conserve and restore river connectivity are gaining momentum. In December 2022, Parties to the Convention on Biological Diversity adopted the Kunming-Montreal Global Biodiversity Framework, which explicitly highlights connectivity in Target 2:“Ensure that by 2030 at least 30 percent of areas of degraded terrestrial, inland water, and coastal and marine ecosystems are under effective restoration, in order to enhance biodiversity and ecosystem functions and services, ecological integrity, and connectivity”(CBD 2022). Despite this growing policy recognition, measuring progress in freshwater connectivity remains a major challenge. Developing effective connectivity indicators is essential to track improvements, guide adaptive management, prioritize restoration efforts, and support watershed-wide conservation plans (McKay et al. 2017).

Among the many connectivity indicators available (reviewed in Jumani et al. (2020)), network-based indicators are widely used because they efficiently represent river networks with relatively little data, enabling analyses at multiple spatial scales to support both local and regional restoration efforts (Erős et al. 2012). One of the most widely used is the Dendritic Connectivity Index (DCI; Cote et al. (2009)), which quantifies longitudinal connectivity by estimating the probability that an individual can move between two reaches based on habitat characteristics and barrier passability. The DCI can be adapted to the movement behaviors of different species, such as potamodromous species ($\mathrm {DCI_p}$), which move entirely within freshwater systems. At the network scale, $\mathrm {DCI_p}$ measures connectivity across the entire watershed, while at the reach scale, $\mathrm {DCI_s}$ assesses connectivity from any reach or segment to the rest of the network (Cote et al. 2009; Mahlum et al. 2014).

A small number of studies have used DCI to evaluate the biological significance of longitudinal connectivity, examining how variation in connectivity relates to fish community structure, species distributions, and abundances. These relationships are context-dependent, as they often depend on habitat quality and vary across species and spatial scales. The most consistent finding across studies is the relationship between longitudinal connectivity (measured by $\mathrm {DCI_p}$) and community structure, with multiple studies reporting a negative correlation between $\mathrm {DCI_p}$ and community dissimilarity (beta diversity; Edge et al. (2017); Mahlum et al. (2014); Perkin et al. (2015); Perkin and Gido (2012). This relationship likely arises because reduced connectivity limits species movement, leading to local species loss and increased dissimilarity among communities. The proportion of variation explained by $\mathrm {DCI_p}$differs considerably, ranging from 5% (Mahlum et al. 2014) to 66% (Perkin and Gido 2012). This disparity likely reflects differences in spatial extent, with smaller-scale studies detecting a stronger relationship between $\mathrm {DCI_p}$ and fish communities due to reduced influence of confounding factors that affect habitat quality. Since environmental factors influencing stream biota are often scale-dependent (Wiens 2002), it follows that the explanatory power of $\mathrm {DCI_p}$ varies with spatial extent. This scale dependence is also evident in the relationship between $\mathrm {DCI_p}$ and species richness. At smaller spatial extents, $\mathrm {DCI_p}$ is positively correlated with species richness, particularly in small watersheds where sampling sites are $<10$ km apart (Perkin and Gido 2012). However, in larger watersheds, this relationship weakens as other factors, such as land cover, exert a stronger influence on community structure (Edge et al. 2017; Mahlum et al. 2014).

Notably, no correlation has been found between the connectivity of individual river segments ($\mathrm {DCI_s}$) and species richness in large watersheds (Edge et al. 2017; Mahlum et al. 2014), suggesting that local abiotic and biotic conditions determine species persistence at individual sites. The relationship between longitudinal connectivity, as measured by DCI, and population abundance and occurrence varies across and within studies, due to differences in species movement behaviors, habitat requirements, and other environmental factors. Some studies report positive correlations between $\mathrm {DCI_s}$ and the abundance of highly mobile species, such as brown trout (*Salmo trutta*) and rainbow trout (*Oncorhynchus mykiss*), suggesting that reduced connectivity, as measured by lower $\mathrm {DCI_s}$ values, may limit movement and reduce local population sizes (Mahlum et al. 2014). However, findings are inconsistent, with some species showing weak or no relationship with $\mathrm {DCI_s}$, likely due to the influence of local habitat conditions, competition, or other ecological constraints. Edge et al. (2017) found no significant association between connectivity and population abundance for common species, apart from longnose dace (*Rhinichthys cataractae*), which exhibited a negative correlation with $\mathrm {DCI_s}$, likely due to confounded habitat preferences rather than a direct effect of connectivity. Patterns in species occurrence further suggest that variation in connectivity, as measured by DCI, is associated with differences in which species persist in fragmented river networks, particularly those with specific life-history traits. Perkin et al. (2015) demonstrated that reduced network-level $\mathrm {DCI_p}$ values were associated with lower occurrence probabilities of pelagic-spawning fishes (i.e., open water spawners), whose reproductive success depends on eggs being transported by currents. Perkin et al. (2015) further showed that species most sensitive to fragmentation were also more vulnerable to flow intermittency, suggesting that multiple environmental factors (e.g. drought) may interact with connectivity loss to shape species distributions. At the reach scale, Mahlum et al. (2014) found that $\mathrm {DCI_s}$ was positively correlated with the occurrence of some species, such as rainbow trout and mottled sculpin (*Cottus bairdii*), but had no significant relationship for others, highlighting that species-specific traits and local habitat conditions influence how connectivity relates to species distributions. Empirical studies have demonstrated a relationship between DCI, as a measure of longitudinal connectivity, and both community structure and population patterns but isolating the effects of connectivity from other environmental factors remains challenging. Direct field assessments of population-level effects, such as changes in abundance, movement patterns, and dispersal rates, require extensive long-term data collection, which is difficult to achieve at the scale of entire river networks. These constraints underscore the need for complementary approaches to evaluate how connectivity, as represented by connectivity indicators, relates to population persistence.

Modelling studies complement empirical research by isolating the influence of connectivity patterns on population dynamics and enabling systematic tests of the predictive power of connectivity indicators across diverse network structures. Many studies have used metapopulation models to examine the role of river network structure in population persistence (Campbell Grant 2011; Carrara et al. 2012; Fagan 2002; González-Ferreras et al. 2019; Lee et al. 2022; Ma et al. 2020; Terui et al. 2018), but they have not been explicitly applied to test the biological relevance of network-based connectivity indicators. Metapopulation theory describes how populations persist in fragmented landscapes by balancing local extinctions and recolonizations Urban and Keitt (2001). Bringing together metapopulation modelling and network connectivity indicators offers a powerful opportunity to test whether indicators like DCI can reliably capture patterns of population persistence in fragmented river networks.

Given the potential of DCI as a biologically meaningful indicator of river connectivity, this study evaluates its ability to predict metapopulation dynamics in fragmented river networks. We used a metapopulation model to simulate the dynamics of a potamodromous fish species, which completes its life cycle entirely within freshwater and depends on connected river networks for dispersal. We assessed the relationship between DCI and persistence in simulated river networks at two spatial scales: (1) at the network scale, where $\mathrm {DCI_p}$ reflects overall longitudinal connectivity across the full river network, and (2) at the reach scale, where $\mathrm {DCI_s}$ quantifies the connectivity of individual river segments. Beyond assessing static network conditions, we also tested whether changes in network-level $\mathrm {DCI_p}$ correspond to changes in metapopulation persistence following barrier removal, providing insight into the reliability of DCI as an indicator for evaluating river connectivity restoration.

We hypothesize that DCI is a strong predictor of metapopulation persistence, as higher connectivity should facilitate dispersal, recolonization, and long-term population viability in fragmented river networks. We do not expect systematically stronger correlations at the network scale than the reach scale, because our study design controlled for confounding variables that might otherwise influence connectivity effects at different spatial scales. We predict that the correlation between DCI and population persistence will be stronger under the following conditions:

- Global dispersal vs. local dispersal, as DCI accounts for barriers across the entire network, whereas populations with local dispersal are primarily influenced by connectivity in their immediate surroundings (Wiersma et al. 2025).
- Density-independent vs. density-dependent metrics, since DCI is derived solely from network structure and does not incorporate population interactions such as local carrying capacity or demographic feedbacks (Cote et al. 2009).
- Branching networks vs. linear networks, because pathways in branching networks allow populations to access other parts of the network despite some barriers, reinforcing the relationship between DCI and persistence. In linear networks even a single low-passability barrier can severely restrict movement, potentially leading to cases where DCI overestimates connectivity relative to actual metapopulation persistence (Fagan 2002).
- Symmetric vs. asymmetric barrier passability, because DCI only considers the product of upstream and downstream passability (Cote et al. 2009), whereas metapopulation colonization rates consider each individually.

Finally, we predict that removing barriers will increase DCI values and lead to corresponding improvements in metapopulation persistence, supporting the utility of DCI as an indicator for river connectivity restoration.

## Methods

### Dendritic Connectivity Index

The dendritic connectivity index (Cote et al. 2009) measures the average probability that an individual can move between two segments in a given dendritic network. Depending on spatial scale and fish life cycle, there are several versions of dendritic connectivity indices. For potadromous fish on the network scale, one defines

1 $$\begin{aligned} \mathrm{{DCI_p} } = \sum _{i=1}^N \sum _{j=1}^N c_{ij} \frac{l_i}{L}\frac{l_j}{L} \times 100, \end{aligned}$$

where *N* is the total number of river segments, $c_{ij}$ is the cumulative passability from segment *j* to segment *i* as defined in Eq. (2), $l_i$ and $l_j$ are the lengths of the segments *i* and *j*, and *L* denotes the total length of the river network. Modifications for anadromous species exist (Cote et al. 2009) but are not of interest to us here.

To calculate the cumulative passability of barriers between segments *j* and *i*, we consider the one and only (and also shortest) path between these two segments. We denote the number of barriers along this path by $K_{ij}$. We multiply the upstream and downstream passabilities ($\alpha _{u,m}, \alpha _{d,m}$) for each barrier *m* along the path and obtain (Cote et al. 2009)

2 $$\begin{aligned} c_{ij} = \prod _{m=1}^{K_{ij}} \alpha _{u,{m}} \alpha _{d,{m}}. \end{aligned}$$

For a localized measure of connectivity, Mahlum et al. (2014) defined the connectivity index of a segment, $\mathrm {DCI_s}$, that measures how connected a given segment is to all other segments. This connectivity index of segment *j* is given by

3 $$\begin{aligned} \mathrm{{DCI_s} }(j) = \sum _{i=1}^N c_{ij} \frac{l_i}{L} \times 100. \end{aligned}$$

Then it is clear that $\mathrm {DCI_p}$ is weighted average of $\mathrm {DCI_s}$ with the relative length of a reach as the weights.

As discussed in Cote et al. (2009) and Mahlum et al. (2014), “length” ($l_i$) need not be the purely geographic length of a reach. If information about habitat suitability is available, “length” can be replaced by any measure of “habitat extent”, or scaled to represent “quality-adjusted length”. For brevity, we will refer to “length,” understood as a proxy for habitat amount and quality. While geographical length is a species-independent quantity, quality-adjusted length depends on the fish species of interest, as does passability.

### Modelling framework

**Fig. 1:**
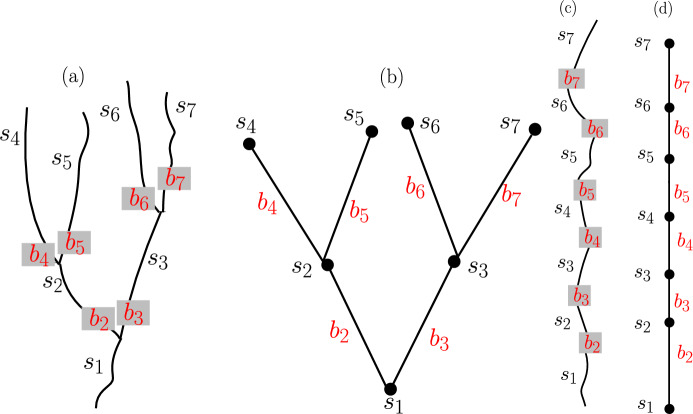
A binary (**a**) and linear (**c**) river network and the corresponding graph representations (**b**) and (**d**), respectively. The water flows from top to bottom. Each river reach or segment, labeled as $s_1,\dots , s_7$ in (a) and (c), is considered a ‘patch’ in the sense of metapopulation models and represented by a solid circle, labeled correspondingly in (b) and (d). Each connection between reaches in (a) and (c), potentially containing barriers labeled $b_2\dots b_7$, is represented as an edge between the corresponding solid circles in (b) and (d) and labeled accordingly. We use breadth-first labeling, starting at the river mouth

To evaluate how well these indices capture population dynamics quantities of interest, such as population persistence and abundance, we simulate model populations on networks of different size and correlate the population dynamic outcomes with the structural connectivity indices. More specifically, we formulate a metapopulation or patch-occupancy model *sensu*Ovaskainen and Hanski (2001) for a potadromous fish species in a watershed, with patches corresponding to stream reaches (Fig. 1). In our model, we allow reaches to be of different (quality-adjusted) length, separated by movement barriers with different passability. The probability of local extinction of an occupied reach decreases with reach length and the probability of colonization of an empty reach depends on the distance to occupied reaches (Ovaskainen and Hanski 2001). In addition, we let the probability of colonization depend on the cumulative barrier passability between the source and target reach, and on their length.

We generate dendritic networks with different numbers of reaches (network size) and randomly chosen reach lengths and barrier passabilities. For each network, we calculate structural indices ($\mathrm {DCI_p}$ and $\mathrm {DCI_s}$) and population-dynamic metrics (metapopulation persistence, long-term patch occupancy), and we compute their correlations on different spatial scales (the network scale, the reach scale) and their sensitivity. We do this for different network topologies and dispersal abilities. We describe each of these aspects in detail in the next section. We structure our results by the spatial scale at which the correlations were calculated (Section “Results”).

### Metapopulation model

We represent a dendritic river network with *N* reaches as a rooted tree graph with each reach corresponding to a node and the mouth of the river (downstream) to the root (Fagan 2002; Rinaldo et al. 2020); see also Dale and Fortin (2010) for a general review of the use of graphs in spatial ecology. Each reach is characterized by its (quality-adjusted) length ($l_i$ with $i=1,\dots N$), i.e., we have a metric graph. An edge in the graph indicates that one reach flows into the other (Fig. 1). To capture the effects of movement barriers (such as culverts or dams), we assign to each edge a *passability* value that indicates the probability of success of moving across this barrier (see Section “Dendritic Connectivity Index”). We denote the passability in the upstream or downstream direction by $\alpha _{u,d}$, respectively. In the absence of barriers, the passability (edge weight) is equal to 1. We only consider stream reaches that are accessible to the species of interest and remove all reaches that are not accessible, for example where there is an insurmountable barrier, i.e., we exclude $\alpha =0$. The resulting tree graph is connected.

Following a general metapopulation or patch-occupancy approach, each reach can be either occupied or empty, and our model tracks the probability of occupancy for each reach over time, $p_i(t)$ with $i=1,\dots N$; see, e.g., Hanski and Ovaskainen (2000) and Ovaskainen and Hanski (2001) for general landscapes and Section 2.4 in Rinaldo et al. (2020) for watersheds, but without consideration of barriers. An occupied reach becomes empty due to a local extinction event whose probability depends on the reach length. An empty reach may become occupied in a colonization event whose probability depends on the distance to other occupied reaches, reach length, intervening barrier passability, and dispersal behavior of the species.

In mathematical terms, the probability of occupancy of reach *i* changes according to the equation

4 $$\begin{aligned} \frac{d p_i(t)}{dt} = \underbrace{C_i(\textbf{p}(t)) [1-p_i(t)]}_\textrm{colonization} - \underbrace{E_i p_i(t)}_\textrm{extinction},\quad \text {for}\quad i=1,\dots ,N, \end{aligned}$$

where $\textbf{p}(t) = (p_i(t), \dots , p_N(t))$ denotes the vector of all occupancy probabilities. The extinction rate of reach *i* decreases with length as

5 $$\begin{aligned} E_i(p(t))=\frac{e}{l_i^{\omega }}, \end{aligned}$$

where *e* is the species-specific extinction parameter and $\omega \in [0,1]$ characterizes the length-extinction relationship. In contrast to the extinction rate, the colonization rate of reach *i* depends on the state of the entire network as

6 $$\begin{aligned} C_i(\textbf{p}(t)) = c ~ l_i^\gamma \sum _{j\ne i} l_j^\epsilon \exp (-d_{ij}/D) ~ \tilde{c}_{ij} ~ p_j(t), \end{aligned}$$

where *c* is the species specific colonization strength. The probability of colonization decreases exponentially with distance “as the fish swims” ($d_{ij}$), relative to the dispersal ability of the species (mean dispersal distance *D*). The number of potential colonizers from reach *j* is $l_j^\epsilon$; hence $\epsilon$ characterizes the length-productivity relationship. Equations (4)–(6) are the standard metapopulation equations (see, e.g., Hanski and Ovaskainen (2000)) with the following minor modification: the colonization rate might depend on habitat length through the term $l_i^\gamma$, with $\gamma$ indicating the length-attractiveness relationship (Wang and Altermatt 2019). When $\gamma =0$, colonization is independent of length.

The cumulative passability, $\tilde{c}_{ij}$, of barriers between source (*j*) and recipient reaches (*i*) must be defined slightly differently here from the expression in (2) because colonization is directional from an occupied to an empty reach. For each barrier along the directed path from one reach to another, we take the passability in the direction that the fish swims; i.e., where a fish passes a barrier in the upstream direction, we consider $\alpha _u$, where it passes in the downstream direction, $\alpha _d$. If we denote the passability of barrier *m* in the same direction as the path by $\alpha _{m, \mathrm dir}$, then the directed cumulative passability is

7 $$\begin{aligned} \tilde{c}_{ij} = \prod _{m=1}^{K_{ij}} \alpha _{m, \mathrm dir}, \end{aligned}$$

where $K_{ij}$ is the number of barriers between the two reaches.

Rinaldo et al. (2020) commented on the plethora of different versions of metapopulation models in the literature. Setting all barrier passabilities equal to one, letting $\omega =\epsilon =1$ and $\gamma =0$, and considering all reaches of the same width so that reach length is proportional to habitat area, our model becomes that of Ovaskainen and Hanski (2001) for terrestrial landscapes. A difference is, however, that a terrestrial “patch” is understood to be suitable habitat for the species, whereas unsuitable areas are usually not considered. By contrast, river reaches of all quality must be included in a dendritic network model because they serve as movement routes. Rinaldo et al. (2020) and others recognize this by considering quality-adjusted length (or area) like we do here.

The dynamics of our model are relatively simple. The extinction state, when all reaches are empty ($\textbf{p}=0$), is a steady state. If it is locally stable, then the metapopulation will face extinction, independent of its initial state. If the extinction state is unstable, then the metapopulation approaches a unique positive state where all reaches are occupied with positive probability ($\textbf{p}^*>0$). These long-term outcomes depend only on the ratio *c*/*e* of the species-specific parameters and not on parameters *c* and *e* individually. The stability of the extinction state can be calculated from the Jacobian matrix (i.e., the linearization of the model at the extinction state). Based on these insights, we define several metapopulation metrics that we then correlate with the aforementioned connectivity indices.

### Metrics of the metapopulation dynamics

We define both network-scale and reach-scale metrics that characterize the metapopulation dynamics and that we correlate with connectivity indices at corresponding scales. We note that $\mathrm {DCI_p}$  is a relative index. When there are no barriers (or all barriers are fully passable), then $\mathrm {DCI_p} =100$. Hence, we consider the metapopulation metrics also in relation to a network without barriers.

#### (i) Metapopulation growth rate

The metapopulation growth rate is the exponential rate of increase or decrease of the number of occupied patches at low occupancy (akin to the population growth rate without density-dependence). It is given by the dominant eigenvalue ($\lambda$) of the Jacobian matrix of model (4) at the extinction state $\textbf{p}=0$. The metapopulation persists if $\lambda >0$ and goes extinct if $\lambda <0$. Hence, it is a network-scale metric. Since the dominant eigenvalue can change sign, whereas $\mathrm {DCI_p}$ does not, it is more appropriate to consider the annualized growth rate $e^{\lambda t}$ with $t=1$ for comparison. We take the ratio

8 $$\begin{aligned} \mathcal{G}_s = \frac{e^{\lambda }}{e^{\lambda _0}} = e^{\lambda -\lambda _0} \end{aligned}$$

as our relative growth metric on the network scale, where $\lambda$ ($\lambda _0$) is the dominant eigenvalue of the Jacobian matrix with (without) barriers.

#### (ii) Basic reproduction number

The basic reproduction number was introduced multiple times in demography, ecology and epidemiology (Heesterbeek 2002). In our context, it measures the average number of occupied reaches that a single occupied reach in an otherwise empty network produces through colonization. Just as the growth rate, this network-scale metric, denoted by $\mathcal{R}$, is also a low-density metric. The metapopulation can persist if $\mathcal{R}>1$ and will go extinct when $\mathcal{R}<1$. We take the ratio

9 $$\begin{aligned} \mathcal{R}_s = \mathcal{R}/\mathcal{R}_0 \end{aligned}$$

as our relative reproduction metric on the network scale, where $\mathcal{R}$ ($\mathcal{R}_0$) is the reproduction number with (without) barriers.

#### (iii) Average steady state occupancy

When the metapopulation persists, all reaches will eventually (for large times) reach a positive occupancy probability, $p^*_i$. We use this to define two additional metrics. A network-scale metric is the average occupancy at steady state, $\bar{p} = (1/N)\sum _{i=1}^N p_i^*$. Hence, we take the ratio

10 $$\begin{aligned} \mathcal{P}_s = \bar{p} / \bar{p}_0, \end{aligned}$$

as our occupancy metric on the network scale, where $\bar{p}$ ($\bar{p}_0$) is the average occupancy with (without) barriers. For the corresponding reach-scale metric, large-time occupancy probability of each reach, $p_i^*$, and divide it by its value in the absence of barriers, $p_{i,0}^*$. We indicate the dependence on each reach *i* by writing

11 $$\begin{aligned} \mathcal{O}_s(i) = p_i^*/p_{i,0}^* \end{aligned}$$

for our occupancy metric on the reach scale.

#### (iv) Reproductive value of a reach

We define a second reach-scale metric of the metapopulation dynamics as the “reproductive value” of a reach. The reproductive value is a well-known concept in matrix population models where it measures the contribution of a given compartment to population growth (Caswell 2000). In our case, it measures the contribution of each reach to the growth in patch occupancy. Formally, it is given by the corresponding entry of the (normalized) left eigenvector of the dominant eigenvalue ($\lambda$) of the Jacobian matrix. More precisely, if $\textbf{v} = (v_1,\dots ,v_N)$ is the left eigenvector to eigenvalue $\lambda$ of the Jacobian matrix at the extinction state $\textbf{p}=0$ (normalized to $\sum _{i=1}^N v_i^2=1$) then the reproductive value of patch *i* is $v_i$. Hence, we take again the relative measure

12 $$\begin{aligned} \mathcal{V}_s(i) = v_i/v_{i,0} \end{aligned}$$

as our reproductive value metric on the reach scale.

In summary, our network-scale metrics for growth, reproduction, and probability of occupancy are $\mathcal{G}_s$, $\mathcal{R}_s$, and $\mathcal{P}_s$, respectively, while our reach-scale metrics of reproductive value and occupancy probability are $\mathcal{V}_s(i)$ and $\mathcal{O}_s(i)$. The metrics $\mathcal{G}_s$, $\mathcal{R}_s$ and $\mathcal{V}_s(i)$ are density-independent persistence metrics whereas $\mathcal{P}_s$ and $\mathcal{O}_s(i)$ are density-dependent steady-state measures.

### Simulations and statistical methods

We generated dendritic networks with randomly chosen (quality-adjusted) reach lengths and barrier passabilities, obtained the corresponding structural indices and the population dynamic metrics on each network, and calculated their correlation coefficients. All computations were done in ‘R’ (R Core Team 2018). Our code is available on a github repository.^1^

We treated network shape as a binary variable and generated linear and full binary networks (R-package igraph) as the two limiting cases of network topology. We expect that results for intermediate binary networks in between these two cases are similar; see Campbell Grant (2011) for a similar approach with a comb-like structure instead of the linear network. Intermediate dendritic networks can be generated with properties that are statistically indistinguishable from real river networks, e.g., optimal channel networks (Rinaldo et al. 2020), so that various continuous characteristics could be chosen instead of our binary ones, but we focus on passability instead. We also treated the dispersal ability of the species as binary, either global dispersal with the average dispersal distance equal to network size ($D=L = \sum _i^N l_i$) or local dispersal with the average dispersal distance equal to the average reach length ($D = L/N$). We fixed the ratio $c/e=10$ as was done in Ovaskainen and Hanski (2001). Since we calculate correlations between relative quantities, the absolute values do not matter.

We consider two different scenarios with respect to barrier passability: (i) symmetric passability, i.e., $\alpha _u = \alpha _d$ for each barrier; (ii) asymmetric passability, where all barriers are completely passable in the downstream direction, i.e., $\alpha _d=1$. We note that the cumulative passability in the DCI ($c_{ij}$ in Eq. 2)) depends only on the product of the upstream and downstream passability at each barrier, whereas the cumulative passability in the metapopulation model ($\tilde{c}_{ij}$ in Eq. 7)) depends on only one passability at each barrier. As a result, the DCI cannot capture asymmetry in upstream and downstream passability, as long as their product is the same, whereas the metapopulation colonization rate reflects asymmetric passability. To allow for comparison between the two scenarios, we pick the upstream passability in the asymmetric case to equal the product of the passabilities in the symmetric case, so that the cumulative connectivity $c_{ij}$ is the same in both cases.

We used Latin hypercube sampling (Stein 1987) with a sample size of 60 to obtain passabilities and lenghts (R-package lhs). Passabilities were chosen from a uniform distribution on (0, 1). We avoided very short reaches by choosing lengths uniformly between half the maximal length and the maximal length. Since the relevant quantities depend only on ratios (see e.g., expressions (1) and (3)), we could set the maximal length to unity. We calculated the $\mathrm {DCI_p}$ and $\mathrm {DCI_s}$ using the R-package fipex. We calculated the Spearman correlation coefficient between the structural indices and the metapopulation metrics. Spearman correlation uses ranked data instead of the numerical values of the data. Calculations of the Pearson coefficient gave qualitatively similar results.

We calculated correlation coefficients on the network scale between $\mathrm {DCI_p}$ and $\mathcal{G}_s$, $\mathcal{R}_s$, and $\mathcal{P}_s$ (see above). We systematically tested these relationships in simulated river networks with a barrier between each pair of reaches. To explore a broad range of conditions, we varied key structural and dispersal parameters across a diverse set of scenarios, including topology (linear vs. branching), network size (3 to 50 reaches), barrier symmetry (symmetric vs. asymmetric passability), and dispersal distance (local vs. global). Both reach length and barrier passability strength were randomized to introduce variation in habitat quality and movement constraints across the networks. On the reach scale, we compared $\mathrm {DCI_s}$ and the local relative metrics occupancy and reproductive value for each reach.

We simulated barrier removal to evaluate whether associated changes in $\mathrm {DCI_p}$ capture changes in metapopulation dynamics. For each barrier in the network, $\mathrm {DCI_p}$ was computed twice, once with the barrier in place and once with it removed (its passability set to unity), and the ratio of the two values was taken as the relative change in structural connectivity. The same procedure was applied to the basic reproduction number ($\mathcal{R}$; see Section “Metrics of the metapopulation dynamics” (ii)) to quantify the metapopulation response to barrier removal. Our choice of sensitivity differs slightly from the one suggested by Pascual-Hortal and Saura (2006), but it is consistent with our earlier definitions of relative metrics. Since the measure proposed by Pascual-Hortal and Saura (2006) is one minus our measure, the resulting correlation coefficients are the same. Results are reported by barrier number (breadth-first numbering) for fixed network sizes.

## Results

### Network-scale metrics and indices

**Fig. 2:**
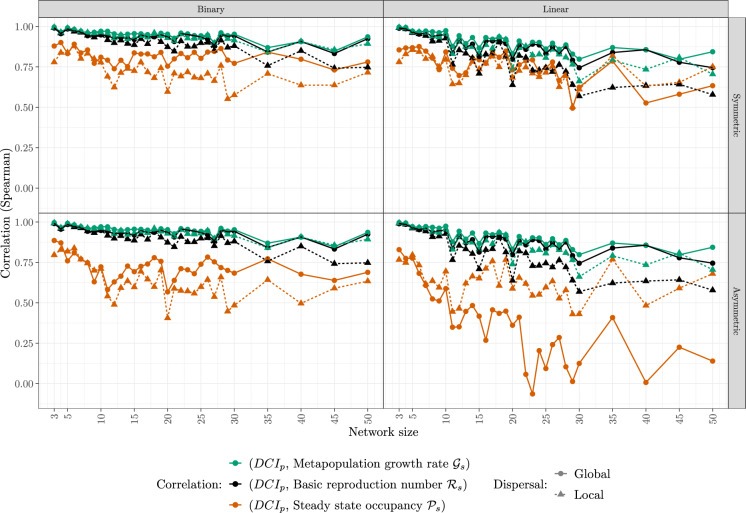
Spearman correlation coefficients between the network-scale index $\mathrm {DCI_p}$ and three metapopulation metrics (see legend for colors) as a function of network size for binary (left) and linear (right) dendritic networks with symmetric (top) and asymmetric (down) barrier passability and global (circle solid) versus local (triangle dashed) dispersal. Parameters are as in Table 1 in the Supplementary Material

Overall, we find strong positive correlations between the network-scale metapopulation metrics and index $\mathrm {DCI_p}$ (Fig. 2), but the details depend on network type (linear vs. binary), dispersal scale (global vs. local) and barrier passability (symmetric vs. asymmetric). In general, we see that the correlations are highest in binary networks with symmetric barrier passability and global dispersal, while they are lowest in linear networks with asymetric passability.

More specifically, the strongest positive correlations occur between $\mathrm {DCI_p}$ and the low-density growth rate $\mathcal{G}_s$ (green) as well as the basic reproduction number $\mathcal{R}_s$ (black) in binary networks with global dispersal (left column plots, circles). The correlations between the same metrics for local dispersal are only marginally lower (left column plots, triangles). Conversely, correlations with the steady-state occupancy $\mathcal{P}_s$ (orange) are generally lower, yet mostly positive except for asymmetric passability and global dispersal in large networks. This suggests that while $\mathrm {DCI_p}$ serves as a reliable predictor for global population metrics, it performs better in forecasting low-density dynamics than capturing density-dependent effects. Since $\mathrm {DCI_p}$ only contains information on structural aspects (geometry, distance, passability), it is to be expected that the effects of population interactions are less well captured.

The correlations are generally higher for global dispersal (circles, solid) than for local dispersal (triangles, dashed), but the correlations with steady-state occupancy $\mathcal{P}_s$ (orange) in linear networks (right column plots) remain an exception. When dispersal is local, the colonization and extinction processes of one reach are largely independent of the conditions far away in the network. Hence, the population metrics show little variation as parameters vary. Yet, $\mathrm {DCI_p}$ does respond to variation further away from a target reach since the effects of barriers and passability are not weighted by distance. Hence, the correlation between the two measures is expected to be lower.

The relationships between correlations and network structure and size are less obvious. Correlations generally appear lower in larger networks, particularly in linear ones. In binary networks, correlations tend to stabilize for low-density metrics as network size increases. However, patterns regarding average occupancy metrics are more erratic, with notable differences between linear and binary networks in large networks. To further explore these patterns, we look at the raw data directly (see Fig. 6 in the Supplementary Material). We observe that with increasing network size, the interquartile ranges of our metrics and indicator decrease considerably (except for $\mathcal{R}_s$). In other words, larger networks share more features and are more alike. When the spread in the data is small, small changes can lead to a change in the rank and hence easily decrease the correlations slightly. At the same time, with the spread being small, there is a high chance that outliers dominate the outcome. To visually test for outliers, we plot the different metrics against our index for network size 40 (see Fig. 7 in the Supplementary Material). We observe that one of the relationships, namely with $\mathcal{P}_s$ is indeed not monotone (see the plot entitled linear:long:Asym:40 in said figure), which corresponds precisely to one apparent outlier in Fig. 2.

The relationship between correlations and barrier (a-)symmetry show some by now familiar patterns. They are highest for small, binary networks with symmetric passability and the low-density metrics $\mathcal{G}_s$ and $\mathcal{R}_s$. By contrast, they are lowest (and in extreme cases even negative) in linear networks with asymmetric passability and the steady-state metric. This demonstrates the importance of asymmetric passability on population processes, an aspect that $\mathrm {DCI_p}$ by its nature cannot capture.

### Reach-scale metrics and indices

**Fig. 3:**
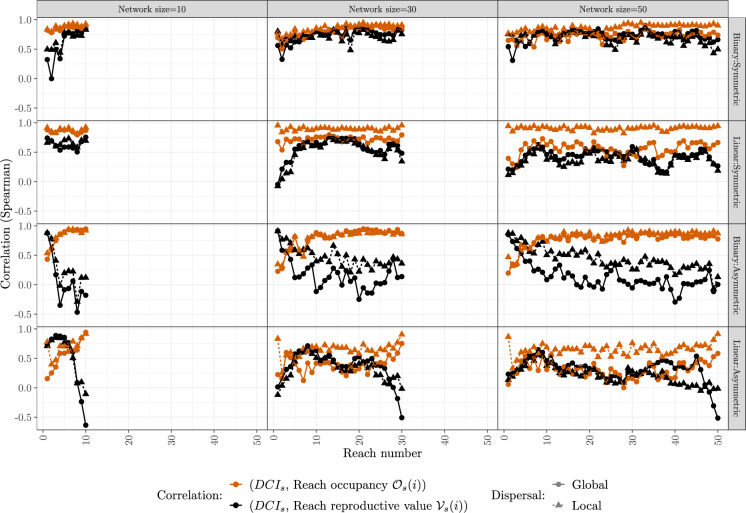
Spearman correlations of local indices in selected networks. For each network size, the correlation coefficients between $\mathrm {DCI_s}$ and reach occupancy $\mathcal{O}_s(i)$ or reproductive value $\mathcal{V}_s(i)$ for reach *i* are calculated for symmetrical and asymmetrical barrier passability. Reaches are numbered from the mouth of the river (reach 1) by a breadth-first numbering as shown in Fig. 1. See Fig. 5 in the Supplementary Material for the plots of other network sizes

On the scale of a single reach (*i*), we correlate $\mathrm {DCI_s}$ with reproductive value ($\mathcal{V}_s(i)$, a low-density metric) and probability of reach occupancy ($\mathcal{O}_s(i)$, a steady-state metric). For a given network size, we calculate the correlation coefficients for each reach and plot them by reach number. We present the results for networks of size 10, 30, and 50 here (Fig. 3); plots for other network sizes are qualitatively similar and can be found in the Supplementary Material (Fig. 5).

While the correlations on the local scale are still quite high overall, some are near zero and some are negative. The overall pattern from the previous section that the correlations are highest in binary networks with symmetric barrier passability is evident here as well (see the top right corner in Fig. 3). While correlations are still positive in linear networks with symmetric passability, asymmetric passability leads to much weaker correlations, in particular for the reproductive value of a patch.

In contrast with the network-scale measures and metrics (Fig. 2), the correlations of $\mathrm {DCI_s}$ with the density-dependent metric (reach occupancy, black) are higher than with the low-density metrics (reproductive values, yellow). Also in contrast with the network-scale measures, the correlations tend to be higher for local dispersal (triangles, dashed) than for global dispersal (circles, solid).

The linear network structure seems to be reflected in the correlations of $\mathrm {DCI_s}$ and reproductive value. Many of the relationships are hump-shaped, i.e., the correlations are lowest at downstream and upstream end of the river.

### Sensitivity of network-scale metrics and indices to barrier removal

**Fig. 4:**
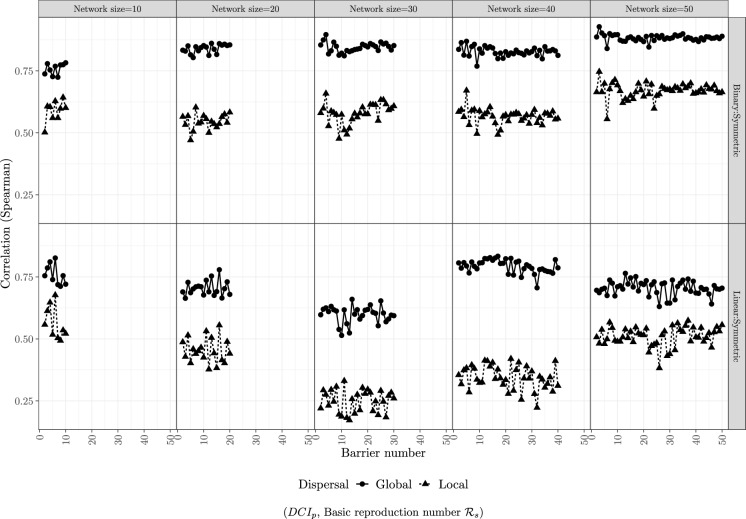
Spearman correlations between the sensitivity of $\mathrm {DCI_p}$ and $\mathcal{R}$ to barrier removal as a function of barrier number for a given network size in the case of symmetric passability. For each generated network and each barrier in that network, we calculated the correlation coefficients between the relative change of the index/metric with respect to removal of this barrier

Correlations between the change in $\mathrm {DCI_p}$ and the basic reproduction number ($\mathcal{R}$) caused by barrier removal are consistently high in binary networks (upper row) with global dispersal (black circles) and lower with short dispersal (black triangles; Fig. 4). The same is true for linear networks (lower row). Correlations tend to be higher in binary than in linear networks (compare upper and lower row) but there are exceptions (e.g. network size 10). Again, we observe some apparent outliers, where the correlations in linear networks seem low (e.g. network size 30). The results in Fig. 4 are for the case of symmetric passability; the correlations for asymmetric dispersal are almost identical since the $\mathrm {DCI_p}$  is independent of whether passability is symmetric and the differences in basic reproduction number between the symmetric and asymmetric cases are negligible (not plotted).

## Discussion

Our simulation study demonstrates a strong correlation between DCI and metapopulation dynamics, reinforcing the value of DCI as an indicator for river connectivity conservation and restoration. More specifically, the results in Section “Network-scale metrics and indices” support our hypothesis that DCI is a strong predictor of global metapopulation persistence metrics, in particular low-density metrics (because DCI does not consider biotic interactions), in a branching network (because no single barrier has undue influence) and under symmetric dispersal (because DCI does not consider asymmetry). Our overall hypothesis that DCI is a good predictor of metapopulation persistence measures is still supported on the scale of a reach, there are some unexpected differences, in particular that the correlations on the local scale are higher with density-dependent metrics and with local dispersal (Section “Reach-scale metrics and indices”). Finally, confirming our hypotheses, we see that the sensitivity of the $\mathrm {DCI_p}$  can be used to prioritize barrier removal since it is highly correlated with the sensitivity of a relevant population-dynamic metric, here the basic reproduction number. This holds in particular in binary networks and for fish species that disperse globally (Section “Sensitivity of network-scale metrics and indices to barrier removal”).

These findings align with previous empirical research demonstrating the biological relevance of DCI, particularly at the scale of the entire river network, where it has been correlated with fish community structure, species distributions, and abundances (Edge et al. 2017; Mahlum et al. 2014; Perkin et al. 2015; Perkin and Gido 2012). While empirical studies have consistently demonstrated the relevance of DCI to network-scale community structure, its relationship to individual species’ population dynamics has been less clear partly due to the difficulty of isolating connectivity effects in field settings and the variability in population responses across species and study systems (Wiersma et al. 2025). Using a simulation-based approach, we examined how DCI correlates with multiple metrics of metapopulation dynamics across a range of fragmentation scenarios, incorporating variation in network topology, barrier configurations, and dispersal abilities. Our results show that $\mathrm {DCI_p}$ reliably predicts network-level metapopulation persistence, measured by growth rate, basic reproduction number, and steady-state occupancy, across a range of river network structures. As expected, DCI is less effective at capturing density-dependent effects, as $\mathrm {DCI_p}$ represents only structural properties (topology, habitat quality, passability) and does not account for population interactions (Cote et al. 2009). Despite this limitation, DCI remained a strong predictor of metapopulation persistence across most scenarios, except for the specific case where linear network topology was combined with asymmetric barrier passability and global dispersal, a combination that likely weakens the expected relationship between connectivity and persistence.

Population persistence at the scale of individual river reaches was also strongly correlated with DCI, supporting the use of DCI as a connectivity indicator for assessing persistence patterns at finer spatial scales. As predicted, correlations between DCI and metapopulation persistence were generally stronger under global dispersal at the network scale, except in linear networks with asymmetric passability, where the relationship weakened. However, at the reach scale, correlations were higher under local dispersal, suggesting that broader network connectivity still contributes to persistence even when movement is restricted. This may reflect the way $\mathrm {DCI_s}$ represents the integration of individual reaches within the broader network, helping to stabilize populations despite limited dispersal distances. Another scale-dependent pattern emerged in the correlation between DCI and density-dependent persistence metrics. While DCI was more strongly correlated with density-independent persistence metrics at the network scale, DCI showed a stronger relationship with the density-dependent persistence metric at the reach scale, a result that warrants further investigation. As expected, network topology influenced correlations, with lower DCI-persistence correlations in linear networks across all scales, where the inherently constrained structure leaves populations more vulnerable to the impacts of barriers (Fagan 2002). Barrier passability symmetry influenced the strength of correlations between DCI and persistence, with generally higher correlations in networks where passability was symmetric. However, the effects of asymmetry were limited and varied by scale and persistence metric. At the network scale, asymmetry weakened correlations only in a specific scenario, while at the reach scale, its effects were more pronounced for density-independent metrics. Given that rivers are inherently unidirectional systems (Peterson et al. 2013), additional research could further explore how barrier asymmetry influences the reliability of DCI across spatial scales and ecological contexts.

Our findings also have direct implications for river restoration planning, particularly for prioritizing barrier removal. A key challenge in river connectivity conservation is determining whether the removal of one or more barriers will meaningfully improve connectivity for fish populations and, if so, which barriers should be prioritized for removal (O’Hanley et al. 2013). Connectivity indices offer a seemingly simple solution-barriers that cause the largest change in the index should be removed first. While previous studies have demonstrated that DCI is sensitive to barrier removal (Cote et al. 2009), our findings extend this by showing that changes in $\mathrm {DCI_p}$ correspond to changes in metapopulation persistence, as measured by the basic reproduction number. This reinforces the value of DCI not only as a structural connectivity indicator but also as a meaningful ecological indicator for assessing the benefits of dam removal. By linking changes in DCI to biologically relevant outcomes, our results strengthen the case for incorporating DCI into connectivity conservation and restoration planning (Erős et al. 2018).

Our results support DCI as a biologically meaningful connectivity indicator, but further validation is needed to assess its applicability across diverse ecological contexts. Empirical studies have linked DCI to species occurrence, abundance, and community composition (Edge et al. 2017; Mahlum et al. 2014; Perkin et al. 2015; Perkin and Gido 2012), but its relationship to population persistence remains less explored. Long-term field studies, such as mark-recapture and genetic analyses, have been used to assess metapopulation dynamics in fragmented streams (e.g., Letcher et al. (2007)) and could provide a framework for testing whether DCI reliably predicts demographic outcomes. Our results suggest that network topology and barrier passability influence how well DCI reflects persistence, with the strongest correlations in branching networks with symmetric passability. Future validation efforts should account for these factors, particularly the degree of asymmetry in barrier passability. While persistence integrates multiple demographic and ecological processes, connectivity can also influence specific aspects of population dynamics, such as access to seasonal resources, reproductive success, and gene flow (Adams et al. 2016; Benitez et al. 2018). Future research should test whether connectivity indicators like DCI are also predictive of variation in these demographic and evolutionary processes. The practical advantages of DCI, its relatively low data requirements and ease of calculation, make it a valuable tool for informing conservation decisions while additional research strengthens its empirical foundations.

Expanding on our findings, future research should test whether the strong correlations between DCI and metapopulation persistence in fragmented river networks hold across a broader range of network topologies, barrier configurations, and ecological complexities. In our simulations, fragmentation was imposed by placing a barrier between each pair of connected reaches, with passability values varying continuously to represent a broad spectrum of movement constraints. Real-world river networks vary widely in the number and distribution of barriers. Assessing the relationship between DCI and metapopulation persistence under a wider variety of fragmentation patterns would help clarify the utility of this index across diverse watershed conditions. Additionally, our model assumed that barrier passabilities were independent, yet in reality, passability may be spatially correlated due to species dispersal abilities, natural hydrological patterns, or management decisions (Cote et al. 2009). Relaxing this assumption could provide a more realistic evaluation of how DCI performs in structured barrier networks. Future research could explore habitat-based restoration by adding high-quality riverine habitat, an approach analogous to patch addition in terrestrial metapopulations (Ovaskainen and Hanski 2003). While our study focused on barrier removal, improving habitat quality within reaches could enhance population persistence by increasing carrying capacity and recolonization potential. Testing how DCI responds to such restoration efforts would further refine its use as a conservation tool. Future studies could extend our findings by using individual-based models to explore how species-specific movement behaviors and life histories influence the relationship between DCI and population persistence. Recent advancements in individual-based models (Day et al. 2023) also allow for the integration of landscape genetics, providing a way to assess how fragmentation and restoration impact genetic diversity and gene flow. Since genetic connectivity is critical for long-term population viability, linking structural connectivity indices like DCI to genetic outcomes would further strengthen their ecological relevance.

Finally, it should be noted that connectivity can result in undesirable negative consequences, such as disease transmission, the spread of invasive species, and even synchronizing local population fluctuations, potentially leading to an increase in extinction risk (Fagan 2002; Earn et al. 2000; Livingstone et al. 2023). In fact, the DCI was recently updated so that practitioners can weigh the benefits of removing freshwater barriers to increase connectivity against the potential for spreading invasive species (Arkilanian et al. accepted).

## Conclusion

Our findings support the use of DCI as an indicator for river connectivity conservation and restoration, given its strong correlation with metapopulation persistence in our simulation study. DCI provides a practical tool for prioritizing dam removal or mitigation efforts, as our results demonstrate that increases in DCI correspond to improvements in metapopulation persistence, supporting the use of this indicator for guiding restoration decisions. As governments and conservation organizations work toward meeting international biodiversity commitments under the Kunming-Montreal Global Biodiversity Framework, reliable and scalable connectivity indicators will be essential for tracking progress on freshwater ecosystem restoration (Target 2). Compared to other connectivity indicators, DCI is relatively easy to measure, requiring fewer data inputs and relying on information that is often more accessible, making it particularly useful for large-scale assessments. However, while our findings reinforce the biological relevance of DCI, further empirical validation is needed across diverse ecosystems and taxonomic groups to ensure its robustness as a conservation indicator.

## Supplementary Information

Below is the link to the electronic supplementary material.

Supplementary file 1 (pdf 1382 KB)

## Data Availability

Our code is available on github: https://github.com/aligharouni/connectivity model.

## Footnotes

1 https://github.com/aligharouni/connectivity_model.
