# Supplementary material for: Connectivity indices can predict population persistence in river networks: insights from a metapopulation model
Source: Landsc Ecol. 2026 Jan 8;41(2):27. doi: 10.1007/s10980-025-02278-8 (PMC12827428; doi:10.1007/s10980-025-02278-8)
Supplement: Supplementary file 1 — (pdf 1382 KB) [file 10980_2025_2278_MOESM1_ESM.pdf]

6 Supplementary Material

In this supplementary material, we provide one table and three additional figures from our numerical explorations.

- Table 1 provides all the parameter values used for the numerical experiments. The code to run the numerical experiments is available on the github repository[2], where ample documentation can be found as well.
- Figure 5 is the extension of Figure 3 from the main text to a wider and finer range of network sizes. Specifically, Figure 5 provides Spearman correlations of local indices for network sizes 5, 10, 15, ... 50, while Figure 3 only showed sizes 10, 30, and 50.
- Figure 6 shows the distribution of values of various metrics rather than only the correlation between them.
- Figure 7 plots each of the metrics against $\mathrm{DCI_p}$for network size 40. We see that all but one relationship is monotone; the outlier is in the fourth plot in the third column under the title 'linear:Long:Asym:40'.

[2]https://github.com/aligharouni/connectivity_model

Symbol	Description	Value
$p_i(t)$	Probability of occupancy of reach i at time t	$[0, 1]$
C_i	Colonization rate of reach i when empty	
E_i	Extinction rate of reach i when occupied	
N	Number of reaches	3–50
c	Species-specific colonization parameter	10
e	Species-specific extinction parameter	1
l_i	(Quality-adjusted) length of reach i (Km) relative to longest reach	$[0.5, 1]$
L	Total length of the network (Km)	$\sum_i l_i$
d_{ij}	Distance between segment j and i (Km)	calculated
D	Mean dispersal distance (Km)	L or L/N
$\alpha_{u,m}$, $\alpha_{d,m}$	Upstream and downstream passabilities of the mth barrier	$(0, 1]$
c_{ij}	Cumulative passability of all barriers from segment j to i	see (2)
$\tilde{c}_{ij}$	Directed cumulative passability of all barriers from segment j to i	see (7)
ω	Length-extinction relationship	1
γ	Length-immigration relationship	1
ϵ	Length-emigration relationship	1

Table 1: Variables and parameters of the metapopulation model specified by equations (4) and illustrated in Figure 1.

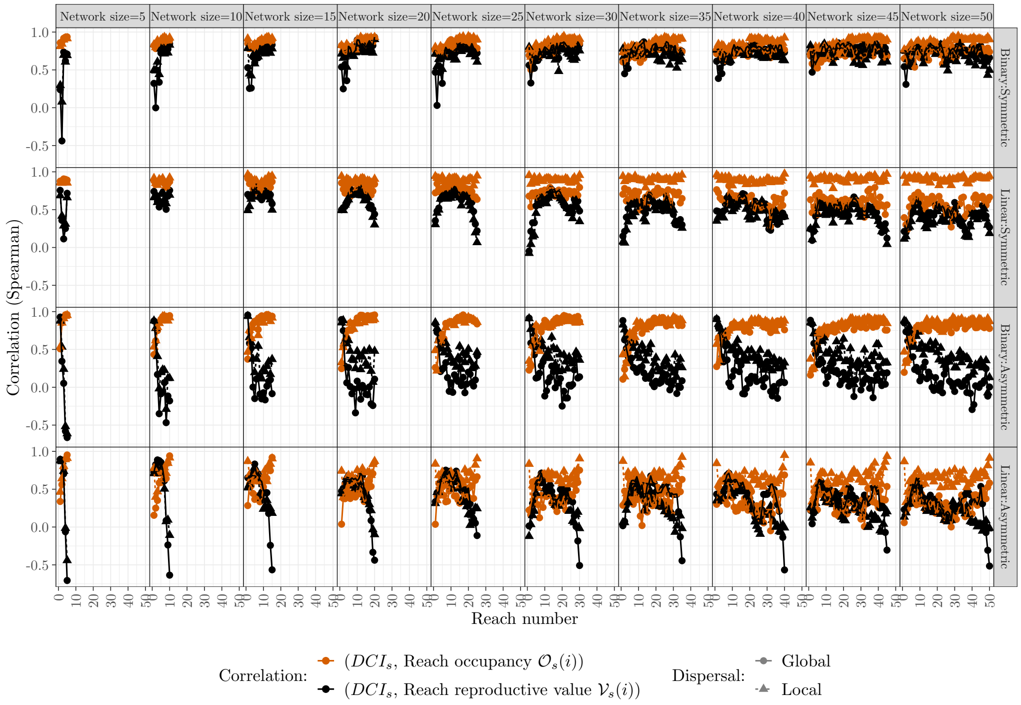

Figure 5: Spearman correlations of local indices in networks. This figure is the extension of Figure 3, where only the data for network sizes 10, 30, and 50 were presented. Just like in Figure 3, for each network size, the correlation coefficients between $\mathrm{DCI_s}$ and reach occupancy or reproductive value are calculated for symmetric and asymmetric passability. Reaches are numbered from the mouth of the river (reach 1) by a breadth-first numbering as shown in Figure 1.

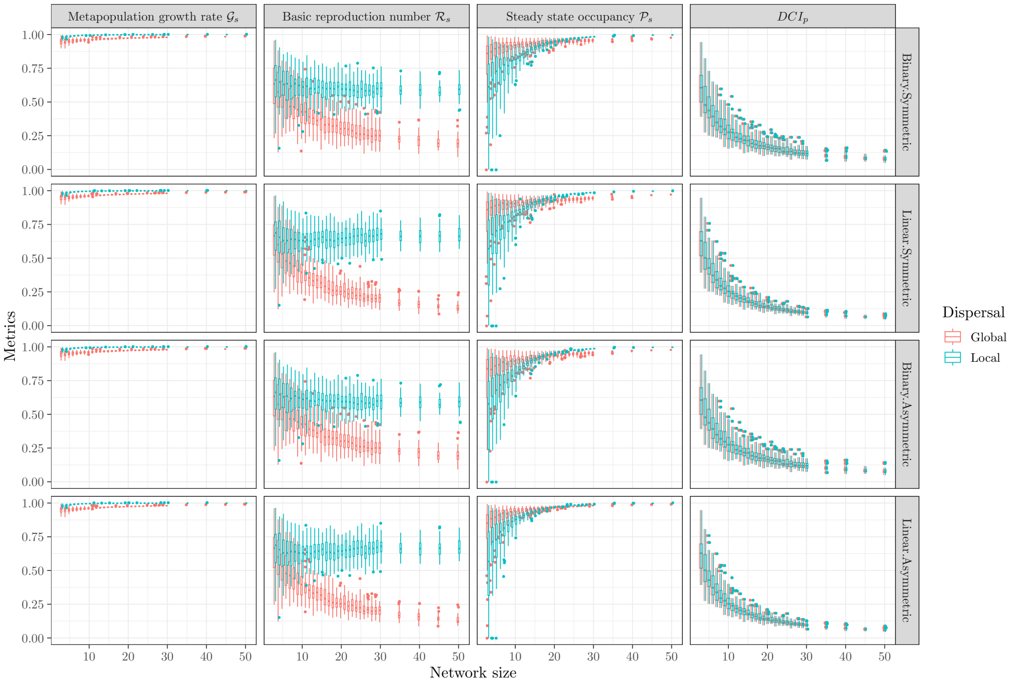

Boxplot shows median (center line), interquartile range (box), and whiskers extending to 1.5×IQR. Outliers are shown as points.

Figure 6: The distribution of the network-scale metrics as a function of network size across grouping factors of network topology and individual movement.

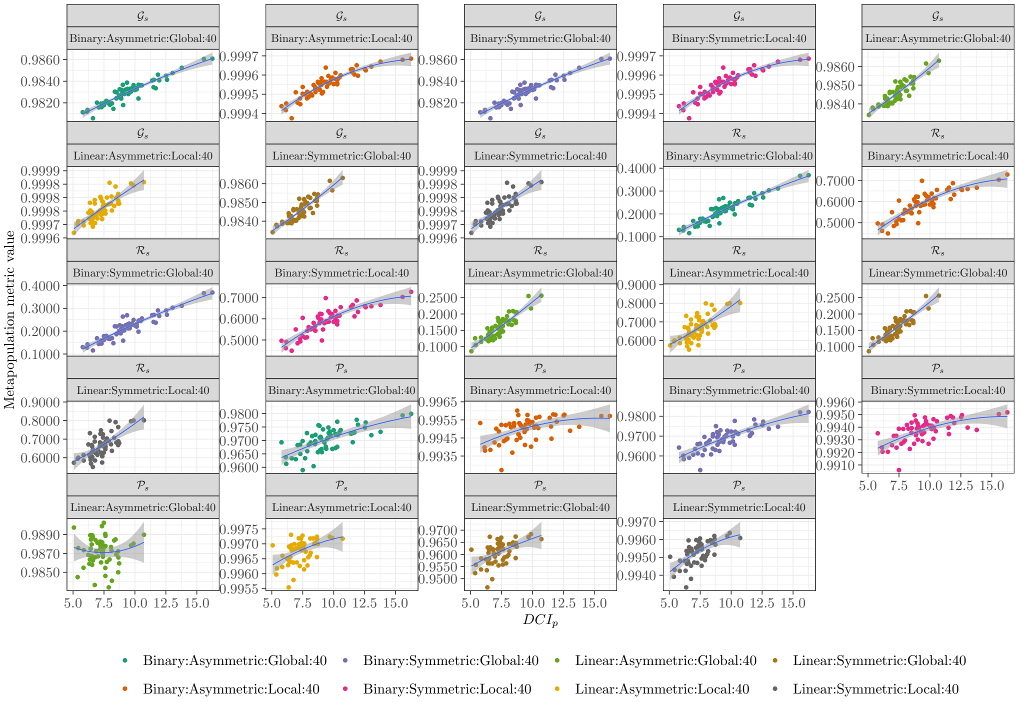

Figure 7: Global metapopulation metrics against $\mathrm{DCI_p}$for network size 40.
